# Performance Evaluation of a HBsAg-Specific Immunoadsorbent Based on a Humanized Anti-HBsAg Monoclonal Antibody

**DOI:** 10.3390/biomedicines13092175

**Published:** 2025-09-05

**Authors:** Shuangshuang Gao, Xiaobin Cai, Tianhui Yan, Yefu Wang, Xinyuan Tao

**Affiliations:** 1 College of Life Sciences, Wuhan University, Wuhan 430072, China; 2 Wuhan Refine Medical Devices Co., Ltd., Wuhan 430070, China

**Keywords:** humanized monoclonal antibody, immunoadsorption, hepatitis B virus (HBV), HBsAg clearance, functional cure

## Abstract

**Background/Objectives**: Hepatitis B virus (HBV) infection poses a major global health challenge, with current therapies like nucleos(t)ide analogs and pegylated interferon alpha offering limited functional cure rates due to persistent HBsAg-driven immune tolerance. This study aimed to develop a targeted immunoadsorption system using a high-affinity humanized anti-HBsAg monoclonal antibody for efficient HBsAg and viral particle clearance, providing a novel approach to overcome therapeutic bottlenecks in chronic hepatitis B (CHB). **Methods**: A murine anti-HBsAg monoclonal antibody was humanized via complementarity-determining region grafting, resulting in HmAb-12 (equilibrium dissociation constant, *K*_D_ = 0.36 nM). A stable Chinese Hamster Ovary K1 (CHO-K1) cell line was established for high-yield expression (fed-batch yield: 8.31 g/L). The antibody was covalently coupled to agarose microspheres (coupling efficiency > 95%) to prepare the immunoadsorbent. Efficacy was evaluated through in vitro dynamic circulation assays with artificial sera and preclinical trials using an integrated blood purification system in two CHB participants. Clearance rates for HBsAg and HBV DNA were quantified, with safety assessed via blood component monitoring. **Results**: In vitro, a single treatment cycle achieved HBsAg clearance rates of 70.14% (high antigen load, >10^5^ IU/mL) and 92.10% (low antigen load, ~3000 IU/mL). Preclinically, one treatment session resulted in acute HBsAg reductions of 78.30% and 74.31% in participants with high and moderate antigen loads, respectively, alongside HBV DNA decreases of 65.66% and 73.55%. Minimal fluctuations in total protein and albumin levels (<15%) confirmed favorable safety profiles, with no serious adverse events observed. **Conclusions**: Preliminary findings from this study indicate that the HBsAg-specific immunoadsorption system can achieve efficient HBV antigen clearance with an initial favorable safety profile in a small cohort. These results support its further investigation as a potential therapeutic strategy for functional cure in CHB. Future work will focus on validating these findings in larger studies and exploring the system’s combinatory potential with existing blood purification platforms.

## 1. Introduction

Hepatitis B virus (HBV) infection continues to pose a major global public health challenge. According to 2021 data from the World Health Organization (WHO), approximately 296 million individuals live with chronic hepatitis B (CHB) worldwide, with a global hepatitis B surface antigen (HBsAg) seroprevalence rate of 3.8%. Additionally, 1.5 million new HBV infections and 820,000 HBV-related liver disease deaths are estimated to occur annually [1]. HBV belongs to the Hepadnaviridae family, a group of small DNA viruses (genome size: ~3.2 kb) that replicate through an RNA intermediate. Its closest relatives include woodchuck hepatitis virus and duck hepatitis B virus, which naturally infect woodchucks and Peking ducks, respectively. Notably, humans are the primary hosts for HBV, though zoonotic transmission to non-human primates has been documented in experimental settings [2]. Structurally, HBV features an envelope with NTCP-binding spikes (HBsAg) and an icosahedral capsid assembled from phosphorylatable HBcAg dimers, both establishing prime targets for neutralizing antibodies and capsid-destabilizing therapies [3]. HBV-transmission occurs predominantly via perinatal exposure, sexual contact, or percutaneous routes involving infected blood/body fluids. Notably, these transmission pathways also facilitate co-infection with hepatitis D virus (HDV)-a defective RNA pathogen that absolutely requires HBV for replication by utilizing HBsAg for viral assembly. This dependency confines HDV infection exclusively to HBV-infected individuals, where superinfection can accelerate liver disease progression [4,5]. Current clinical management primarily relies on combination therapy with nucleos(t)ide analogs (NAs) and pegylated interferon alpha (PEG-IFNα). However, both approaches exhibit significant limitations in controlling persistent viral infection [6].

Mechanistically, NAs exert their antiviral effect by inhibiting viral DNA polymerase activity but fail to directly target the covalently closed circular DNA (cccDNA), the core template for HBV replication within hepatocytes [7,8]. Due to the long-term persistence of cccDNA and the integration of the HBV genome into host hepatocytes, NA therapy only achieves a slow decline in HBsAg levels. For patients with HBsAg > 1000 IU/mL, the HBsAg seroclearance rate is as low as 1.4%. Furthermore, long-term NA use carries risks of drug resistance and potential severe hepatitis flare-ups upon discontinuation [9,10,11]. PEG-IFNα suppresses HBV transcription and replication by modulating innate immune mechanisms and the direct antiviral actions of interferon-stimulated genes, achieving a long-term HBsAg loss rate of approximately 20% at 5 years post-treatment [12,13,14]. However, PEG-IFNα therapy is frequently associated with adverse effects such as flu-like symptoms, leukopenia, and thrombocytopenia, which exhibit a high incidence rate [15]. Therefore, novel therapeutic strategies urgently need to be developed for HBV.

Current studies have reached a consensus that HBsAg overload-induced host immune tolerance is a core barrier to achieving a functional cure for HBV [16,17]. This HBsAg overload originates not only from intact virions but also, predominantly, from a sizable excess of immature, genome-free subviral particles (HBsAg particles). Early studies found that over 67% of virions secreted in vitro are empty shells, while in the host bloodstream, this proportion is even higher, often exceeding 97% [18]. Recent research further confirms that serum levels of empty viral particles vastly outnumber those of DNA-containing virions during HBV infection, typically by more than 100-fold, and can reach tens of thousands of times higher in some cases [19,20]. In this context, neutralizing antibodies targeting HBsAg offer a promising strategy. They can reduce antigen load not only through Fcγ receptor-mediated clearance of viral particles but also by restoring the function of HBV-specific CD8^+^ T cells, thereby potentially breaking immune tolerance [21,22,23]. Antibody engineering advancements based on structural biology, particularly the introduction of pH-dependent antigen-binding domains and FcRn-binding-enhancing mutations, have demonstrated simultaneous clearance of serum and intrahepatic HBsAg in mouse models, inducing antiviral T/B cell memory responses [24].

In recent years, immunoadsorption therapy based on neutralizing antibodies has emerged as a research focus due to its high specificity and excellent reproducibility. This technology utilizes immobilized anti-HBsAg antibodies to specifically capture circulating HBsAg and intact virions. in vitro studies indicate its potential for rapid viral load reduction [25,26]. However, existing adsorption systems often rely on blood-derived human hepatitis B virus immunoglobulin or murine anti-HBsAg monoclonal antibodies (anti-HBsAg mAbs), presenting technical bottlenecks such as high costs, an insufficient capacity for dynamic adsorption, and potential immunogenicity risks. Furthermore, current research is largely confined to animal model testing, necessitating systematic evaluation of engineered antibody expression, clinical application safety, and efficacy.

To address these key scientific challenges, this study successfully obtained a high-affinity murine anti-HBsAg mAb. We performed antibody humanization via complementarity-determining region (CDR) grafting and established a high-expressing stable CHO-K1 (Chinese Hamster Ovary K1) cell line (with its fed-batch yield reaching 8.31 g/L). Building upon this, we successfully developed a novel HBsAg-specific immunoadsorption system. Our major breakthroughs are as follows: (1) The humanized anti-HBsAg mAb HmAb-12 exhibits high affinity for HBsAg (*K*_D_ = 0.36 nM). (2) Dynamic circulation adsorption experiments demonstrated that a single cycle achieves a 70.14% clearance rate of HBsAg from sera with high viral loads (>10^5^ IU/mL). (3) Preclinical trials confirmed that a single treatment session achieves approximately 75% HBsAg clearance in human blood. This technological platform provides an innovative solution characterized by high efficiency and safety, aiming to overcome the current bottlenecks in CHB therapy. Its clinical translation holds promise for reshaping existing treatment strategies.

## 2. Materials and Methods

### 2.1. Preparation and Characterization of Murine Anti-HBsAg mAb

The procedure was adapted from Huang et al. [27] with modifications. This study, based on the widely accepted “3Rs” principle, optimized the design and strictly planned the number of animals required. All mouse experiments were performed in the Experimental Animal Center of Huazhong Agricultural University (Wuhan, China) with approval from the institution’s Scientific Ethics Committee (Ethics Approval No.: HZAUMO-2020-185). Eight-week-old female BALB/c mice were immunized. The primary immunization used recombinant HBsAg (Wuhan Yuening Technology Co., Ltd., Wuhan, China) at a dose of 100 μg per mouse. A booster immunization was administered identically on day 15. On day 30, tail vein blood was collected, and the serum titer was assessed using an indirect enzyme-linked immunosorbent assay (iELISA). Mice exhibiting titers ≥ 1:32,000 were selected for cell fusion.

BALB/c mouse peritoneal macrophages served as feeder cells. A suspension of SP2/0 myeloma cells (Cat# CL-0445, Wuhan Pricella Biotechnology, Wuhan, China) was prepared from BALB/c mice, and a spleen cell suspension was obtained from an immunized BALB/c mouse. Myeloma cells (SP2/0 lineage) were employed as fusion partners due to their immortal proliferative capacity, which enables stable hybridoma generation and continuous antibody secretion post-fusion with antigen-primed splenocytes [28]. Immune splenocytes and SP2/0 myeloma cells were mixed at a 2:1 ratio. Fusion was induced by adding 50% PEG (pH 8.0) for 30 s, followed by the addition of incomplete DMEM to terminate the reaction after 1 min. The cells were centrifuged, resuspended in 5 mL of 2% HAT-supplemented complete DMEM, diluted into 120 mL of HAT-supplemented complete DMEM, and plated onto 96-well plates pre-seeded with feeder cells. The plates were incubated in a CO_2_ incubator. When the cell colonies reached sufficient size, the culture supernatants were screened for positive clones via iELISA. Positive hybridoma clones were subcloned using the limiting dilution method [29].

Antibody-producing monoclonal hybridoma cells (1 × 10^6^ cells per mouse) were intraperitoneally injected into the BALB/c mice. Ascitic fluid was collected 5–10 days post-injection and stored at −80 °C. The antibodies were initially enriched from ascites using caprylic acid-ammonium sulfate precipitation, followed by purification via Protein A affinity chromatography. The purified antibodies were then characterized using sodium dodecyl sulfate–polyacrylamide gel electrophoresis (SDS-PAGE) and Western blotting (WB).

The antibody titer was determined using a commercial Diagnostic Kit for Antibody to HBsAg (ELISA) (Shanghai Kehua Bio-Engineering Co., Ltd., Shanghai, China). Antibody isotyping was performed according to the manufacturer’s instructions for the Mouse mAb Isotyping Kit (Cat# PK20003, Proteintech, Wuhan, China).

### 2.2. SDS-PAGE

SDS-PAGE was employed to analyze the antibody molecular weight and purity. The protein samples were mixed with 5× SDS loading buffer and denatured by heating at 95 °C for 15 min in a metal bath. After centrifugation, the supernatant was collected for loading. Separation was performed using 4–15% precast gradient gels (Sangon Biotech, Shanghai, China). Following electrophoresis, the gels were stained with Coomassie Brilliant Blue staining solution (Biosharp Life Sciences, Hefei, China) on a shaker for 30–60 min. The gels were destained with multiple destaining solutions until the backgrounds were clear and images were captured.

### 2.3. WB

A 20 μL aliquot of the murine mAb sample was mixed with 80 μL of a 5× SDS loading buffer and denatured at 95 °C for 10 min. A 10% separating gel was prepared. Ten microliters of the denatured sample was loaded per well and separated via SDS-PAGE. After disassembling the gel, the stacking gel was removed and the separating gel was rinsed with deionized water. A PVDF membrane, pre-activated with methanol, was assembled into the transfer cassette in accordance with the following sequence: filter paper, PVDF membrane, gel, and filter paper. The transfer was performed at 24 V constant voltage for 60 min in the transfer buffer. Post-transfer, the PVDF membrane was blocked with TBST containing 5% skimmed milk powder for 30 min at room temperature. The membrane was washed three times with TBST for 5 min each. Horseradish peroxidase (HRP)-conjugated goat anti-mouse IgG antibody (Cat# AS003, ABclonal, Wuhan, China; diluted 1:5000 in blocking buffer) was added and incubated for 60 min at room temperature. The membrane was washed three times with TBST for 5 min each. An ECL chemiluminescent substrate (Cat# PR1200, Solarbio, Beijing, China) was added, and the membrane was developed using an automatic film processor.

### 2.4. Construction of Humanized mAb

Total RNA was extracted from hybridoma cells using the Cell Total RNA Isolation Kit (Cat# RE-03111, FOREGENE, Chengdu, China). First-strand cDNA was synthesized from total RNA using the ReverTra Ace-α-First Strand cDNA Synthesis Kit (Cat# FSK-100, TOYOBO, Osaka, Japan). The variable heavy (VH) and light (VL) chain gene fragments of the murine mAb were amplified via PCR, sequenced, and analyzed.

The variable region sequences (VH and VL) of the murine antibody were cloned into the eukaryotic expression vector pTT5 containing the constant domains of human immunoglobulin to construct chimeric antibody expression plasmids pTT5-VH and pTT5-VL, respectively. The HEK293E suspension cells were transiently co-transfected with pTT5-VH and pTT5-VL plasmids using polyethylenimine (PEI). The culture supernatants were collected, and chimeric antibodies were purified using Protein A affinity chromatography. The half-maximal effective concentration (EC_50_) for HBsAg binding was determined using iELISA.

CDRs were identified using the IMGT numbering scheme. Homology modeling, CDR grafting, and optimization of key framework residues via point mutations were performed using the Molecular Operating Environment (MOE 2019.0102) software and antibody structural databases. This resulted in four humanized VH sequences (HVH1-HVH4) and three humanized VL sequences (HVL1-HVL3). These were combinatorially paired to generate 12 humanized antibody variants, designated HmAb-1 to HmAb-12, each comprising humanized variable domains and human immunoglobulin constant domains.

### 2.5. CHO-K1 Cell Transfection and Stable Pool Generation

The amino acid sequences of the humanized antibody heavy- and light-chain variable regions were codon-optimized, and the optimized gene sequences were synthesized. The optimized heavy- and light-chain variable gene fragments were cloned into the eukaryotic expression vector using HindIII (NEB) and XhoI (NEB) restriction sites, respectively.

CHO-K1 cells (Cat# 13080801, the European Collection of Authenticated Cell Cultures, Porton Down, UK) were resuscitated and cultured in a complete medium HyClone^TM^ HyCell^TM^ TransFx-C medium (Cat# SH30941.02, Cytiva, Logan, UT, USA) supplemented with 4 mM L-glutamine (Cat# A2916801, Gibco, São Paulo, Brazil) and 0.1% Pluronic^TM^ F-68 (Cat# 24040-032, Gibco, Grand Island, NE, USA). After several shake flask passages, the cells were seeded at a density of 7 × 10^5^ cells/mL one day prior to transfection. Transfection was performed via electroporation (Gene Pulser Xcell, Bio-Rad, Hercules, CA, USA). For each transfection, 1 × 10^7^ cells were transfected with 20 μg of light chain plasmid and 20 μg of heavy chain plasmid. The electroporation parameters were 300 V and 950 μF (exponential decay pulse). Post-transfection, the cells were seeded into T25 flasks at a density of 2–5 × 10^5^ cells/mL. After 48 h, the medium was replaced with a selection medium (HyClone^TM^ HyCell^TM^ TransFx-C medium + 0.1% Pluronic^TM^ F-68 + 25 μM methionine sulfoximine (MSX) (Cat# M5379, Sigma, Saint Louis, MI, USA) + 20 μg/mL puromycin (Cat# ant-pr-1, InvivoGen, San Diego, CA, USA). The cells were cultured statically at 36.5 °C, 80% humidity, and 5% CO_2_. Cell density and viability were monitored every 3–6 days. When cell density exceeded 1 × 10^6^ cells/mL and viability was >70%, the cultures were switched from static to agitated conditions. The cultures were maintained until cell density > 1 × 10^6^ cells/mL and viability > 90%, at which point the stable pool was considered established.

### 2.6. Monoclonal Cell Line Cloning and Screening

The stable pool was resuscitated and passaged several times. The EX-CELL^®^ CHO cloning medium (Cat# C6366, Merck, Saint Louis, MI, USA) was dispensed (100 μL/well) into 96-well plates. A single-cell printer (C.sight 2.0, Cytena, Cytena, Germany, Germany) was used to seed cells at a density of one cell per well. The plates were cultured statically at 36.5 °C, 5% CO_2_, and 80% humidity. Clonality was confirmed using a cell imager (Cell Metric, Solentim, Wimborne, UK) on days 0, 1, 2, and 8 post-seeding. After imaging on day 8, 100 μL of HyClone^TM^ HyCell^TM^ CHO cell culture media (Cytiva) was added to each well for clone expansion, and the static culture was continued.

On day 20, the antibody titer in the culture supernatant was measured using a molecular interaction analyzer (Octet Qke, ForteBio, Fremont, CA, USA). High-producing clones were selected based on the titer and single-cell imaging results and transferred to 24-well deep-well plates for expansion. The titers were re-assessed after expansion in the deep-well plates. Based on the titer results, the highest-producing clones were transferred to 50 mL TPP TubeSpin^®^ bioreactors (Cat# 87050, TPP, Trasadingen, Switzerland) for culture.

### 2.7. Fed-Batch Culture of Monoclonal Cell Lines

Selected monoclonal clones were inoculated into 50 mL TPP TubeSpin^®^ bioreactors for fed-batch culture. The working volume was 20 mL, with an initial seeding density of 1 × 10^6^ cells/mL. The basal medium was HyClone^TM^ HyCell^TM^ CHO cell culture media supplemented with 0.1% Pluronic^TM^ F-68. The culture conditions were a temperature of 36.5 °C, humidity of 85%, CO_2_ 5%, and an agitation speed of 220 rpm. The feeding strategy was as follows: On day 0 (inoculation), 5% (*v*/*v*) HyClone^TM^ Cell Boost^TM^ 5 Supplement (Cytiva), 100 ng/mL recombinant human LR3 insulin-like growth factor-1 (rHuLR3 IGF-1) (PrimeGene, Shanghai, China), and 1 μM MnCl_2_ (Sigma) were added. On days 3 and 5, 5% (*v*/*v*) MaxFX Feed Medium (MediumBank, Shanghai, China) and 0.5% (*v*/*v*) MaxFB Feed Medium (MediumBank, Shanghai, China) were added. On day 7, 50 ng/mL rHuLR3 IGF-1 and 1 μM MnCl_2_ were added. On days 7, 9, 11, 13, 15, and 17, 3% (*v*/*v*) MaxFX Feed Medium and 0.3% (*v*/*v*) MaxFB Feed Medium were added. The temperature was shifted to 32 °C on day 4. Glucose was supplemented to a final concentration of 6 g/L whenever the concentration in the culture dropped below 4 g/L. The cultures were harvested on day 17 or when cell viability dropped below 60%.

The top five monoclonal cell lines (Top 5) were selected based on their antibody expression levels and cell growth data. The fed-batch culture supernatants from the Top 5 clones were purified using Protein A affinity chromatography. The purified antibody products underwent quality analysis, including size-exclusion chromatography-high-performance liquid chromatography (SEC-HPLC) for purity and capillary electrophoresis-sodium dodecyl sulfate (CE-SDS) for purity assessment.

### 2.8. Cell Density and Viability Measurement

CHO cell density and viability were determined using the trypan blue exclusion assay. One milliliter of CHO cell suspension was centrifuged at 300× *g* for 3–5 min. The supernatant was discarded, and the pellet was resuspended in 2 mL of Dulbecco’s phosphate-buffered saline. An equal volume (100 μL) of 0.4% trypan blue solution was mixed with 100 μL of the CHO cell suspension. Fifteen microliters of the mixture was loaded into a cell counting chamber, and the total cell density and viable cell density were measured using an automated cell counter.

### 2.9. Antibody Binding Activity Assay

The binding activity of the antibody to HBsAg was evaluated by determining the EC_50_ using an iELISA as follows: A recombinant HBsAg antigen (0.5 μg/mL in PBS, 100 μL/well) was coated onto 96-well plates overnight at 2–8 °C. The wells were washed three times with 200 μL of PBST (PBS + 0.05% Tween-20), with 1 min incubation per wash. The plates were blocked with 200 μL of 5% bovine serum albumin (BSA) in PBS for 2 h at 37 °C. After blocking, the wells were washed three times with PBST. Serially diluted test antibodies (100 μL/well) were added and incubated for 1.5 h at 37 °C; PBST served as the negative control. The wells were washed three times with PBST. HRP-conjugated secondary antibodies were added (100 μL/well): HRP-conjugated horse anti-mouse IgG antibody (Cat# 7076S, Cell Signaling Technology, Danvers, MA, USA) for murine antibodies; HRP-conjugated donkey anti-human IgG antibody (Cat# SA00001-11, Proteintech, Wuhan, China; diluted 1:5000 in blocking buffer) for humanized/chimeric antibodies. The plates were incubated for 1 h at 37 °C. After incubation, the wells were washed three times with PBST. A TMB substrate (Cat# PR1200, Solarbio, Beijing, China) was added for color development. The reaction was stopped by adding 100 μL of 2 M H_2_SO_4_ after maximum color development. Absorbance was measured at 450 nm using a microplate reader. Dose–response curves were fitted, and EC_50_ values were calculated using GraphPad Prism 8.0 software.

### 2.10. Antibody Affinity Measurement

The equilibrium dissociation constant (*K*_D_) for antibody binding to HBsAg was determined using surface plasmon resonance (SPR) on a Biacore^TM^ 8K system (Cytiva). The experimental setup was as follows: sensor chip, CM5; running buffer, 10 mM HEPES (pH 7.4), 150 mM NaCl, 3 mM EDTA, and 0.05% Tween 20; and analysis temperature, 25 °C. The recombinant HBsAg antigen was covalently immobilized onto the chip surface via amine coupling. The test antibody samples were serially diluted in the running buffer to eight concentrations (100 nM, 50 nM, 25 nM, 12.5 nM, 6.25 nM, 3.125 nM, 1.5625 nM, and 0.78125 nM). Binding kinetics were assessed using a single-cycle kinetics mode, with a flow rate of 30 μL/min, an association time of 120 s, and a dissociation time of 300 s. After each binding/dissociation cycle, the chip surface was regenerated with 10 mM glycine-HCl (pH 1.5) at 30 μL/min for 30 s. Sensorgrams were analyzed using Biacore^TM^ Insight Evaluation Software (Version 5.0.18.22102). A 1:1 Langmuir binding model was applied to calculate the association rate constant (ka), dissociation rate constant (kd), and *K*_D_ (*K*_D_ = kd/ka).

### 2.11. SEC-HPLC and CE-SDS Analysis Methods

Size-Exclusion Chromatograph–High-Performance Liquid Chromatography (SEC-HPLC): Purified antibody samples (100 μg/mL) were analyzed using a ZENIX SEC-300 column (SEPAX, Newark, NJ, USA) on an Agilent 1290 HPLC system (Agilent, Santa Clara, CA, USA). The mobile phase was 100 mM phosphate buffer (pH 7.0) at a flow rate of 0.5 mL/min. Absorbance was monitored at 280 nm to assess the monomer purity and the content of high-molecular-weight and low-molecular-weight species.

Capillary Electrophoresis-Sodium Dodecyl Sulfate (CE-SDS): The analysis was performed using an PA800 Plus Pharmaceutical Analysis System (SCIEX, Framingham, MA, USA) with the SDS-MW Analysis Kit. This kit utilizes a replaceable gel matrix to separate protein-SDS complexes (effective separation range ~10–225 kDa). For reduced samples, 95 μL of the antibody sample (0.5 mg/mL) was mixed with 5 μL of 2-mercaptoethanol and 2 μL of the internal standard and then heated at 100 °C for 3 min. The processed samples were analyzed following the PA 800 Plus system operating manual. Molecular weight standards were used to estimate the apparent molecular weights of the protein components in the sample.

### 2.12. Immunoadsorbent Preparation, Antibody Coupling Efficiency, and Clearance Rate Calculation

An HBsAg-specific immunoadsorbent was prepared using the sodium periodate oxidation method, adapted from Zhang et al. [30]. Focurose HPL agarose microspheres (diameter 45–165 μm) served as the matrix, and the humanized anti-HBsAg mAb HmAb-12 served as the ligand. One gram of Focurose HPL agarose microspheres was conjugated with the corresponding amount of mAb. The antibody concentrations before and after conjugation were measured using a Bradford assay kit (Cat# PC0010, Solarbio, Beijing, China).

The antibody coupling efficiency (CO_E_, %) was calculated using Equation (1):

CO_E_ = (C_1_ − C_2_)/C_1_ × 100% (1)

where C_1_ = initial antibody concentration (mg/mL) and C_2_ = residual antibody concentration in the flow-through after coupling (mg/mL).

The HBsAg clearance efficiency (CL_E_, %) for the adsorbent was calculated using Equation (2):

CL_E_ = (C_0_ − C)/C_0_ × 100% (2)

where C_0_ = initial HBsAg concentration (IU/mL) and C = residual HBsAg concentration after adsorption (IU/mL). The HBsAg concentrations were determined using a chemiluminescent immunoassay.

### 2.13. Immunoadsorbent Characterization and Evaluation

Scanning Electron Microscopy (SEM) Analysis: Agarose microspheres were uniformly dispersed in absolute ethanol and sonicated for 5 min. A 10 μL aliquot of the suspension was dropped onto a clean glass coverslip and dried in a 37 °C vacuum oven. The dried sample was mounted on conductive carbon tape and sputter-coated with a gold/platinum alloy layer using an ion sputter coater (Model EM ACE200, Leica Microsystems, Wetzlar, Germany; coating time 20 s). The surface and cross-sectional morphologies were observed using a field emission scanning electron microscope (Model SU8100, Hitachi High-Tech Corporation, Tokyo, Japan) at an accelerating voltage of 3 kV and a working distance of 8–10 mm.

Cytotoxicity Test (MTT Assay): Performed according to the Chinese National Standard GB/T 16886.5-2017 (Biological evaluation of medical devices—Part 5: Tests for in vitro cytotoxicity) [31], the adsorbent extract (prepared per the standard) was co-incubated with mouse fibroblast cells (L929). The cell relative viability was assessed using the MTT assay to evaluate potential cytotoxicity.

Static Adsorption Assay: A total of 0.2 g of the adsorbent was mixed with 2 mL of an artificial HBsAg-positive bovine serum (prepared by spiking recombinant HBsAg antigen into bovine serum at predetermined concentrations) in a centrifuge tube. The mixture was incubated at 37 °C with shaking for 10 min. After settling for 30 min, the supernatant was collected and the residual HBsAg concentration was measured.

Effect of Storage Solutions: To evaluate the impacts of different storage solutions on long-term adsorbent stability, the freshly prepared adsorbent was resuspended in either 0.4 g/L polyhexamethylene biguanide hydrochloride (PHMB) solution, 20% (*v*/*v*) ethanol solution, or 1 g/L sodium azide (NaN_3_) solution. After storage at 4 °C for 30 days, static adsorption assays were performed to measure the HBsAg clearance rates.

Accelerated Stability Testing: An adsorbent stored in the selected solution (1 g/L NaN_3_) was subjected to accelerated aging in a 37 °C incubator. The samples were taken at days 0 (freshly prepared adsorbent, equilibrated to 37 °C in the storage solution), 2, 4, 10, and 30. The HBsAg clearance rates were determined via static adsorption assays.

Dynamic Adsorption Assay: A total of 3.6 g of the adsorbent was packed into a 5 mL pre-packed chromatography column. The column was equilibrated with 10 column volumes of physiological saline. Subsequently, 50 mL of artificial HBsAg-positive bovine serum (with different initial HBsAg concentrations: low-load group’s baseline = 3000 IU/mL; high-load group’s baseline = 100,000 IU/mL) was perfused through the column in a top-down recirculation mode at a flow rate of 10 mL/min. The total recirculation time was 40 min (each 10 min interval was considered one cycle). Effluent serum samples were collected at the end of each cycle (i.e., 10 min, 20 min, 30 min, and 40 min) for residual HBsAg concentration measurement and clearance rate calculation.

### 2.14. Trial Design

This prospective, single-center preclinical feasibility trial (ethics approval No.: 20230206) was conducted at Wuhan Kane Hospital (Wuhan, China). An extracorporeal plasma purification system was employed, consisting of a plasma separator (Model PE-08, Asahi Kasei Medical Co., Ltd., Tokyo, Japan) connected in series with the developed HBsAg-specific immunoadsorption column (Model HBV-250, Wuhan Refine Medical Devices Co., Ltd., Wuhan, China; column volume, 250 mL; ligand density, 4 mg antibody/g microspheres). Two eligible CHB participants underwent a single extracorporeal purification treatment each.

Treatment Procedure: The immunoadsorption column and plasma separator were primed according to the manufacturer’s instructions. An extracorporeal circulation circuit was established via central venous catheterization, connecting a blood pump (main line) and a plasma pump (branch line), followed sequentially by the plasma separator, a bilirubin adsorption column, and the HBsAg-specific immunoadsorption column. Circuit integrity was confirmed. The blood flow rate was 100–120 mL/min; the target processed plasma volume was 1.8–2.5 L; and purified plasma was returned to the participant. Sampling and Testing: Venous blood was collected immediately before treatment initiation, at the end of treatment (±5 min), and at 24 ± 4 h post-treatment. The tested parameters included HBsAg concentration, HBV DNA load, complete blood count, and blood biochemistry (focusing on total protein [TP] and albumin [ALB]). Treatment Termination: Upon reaching the target plasma volume, the plasma pump was stopped first, followed by the blood pump. The arterial line was disconnected, and residual blood in the circuit was returned to the participant using saline. The circuit was disconnected, ending the treatment. The evaluation metrics included the acute HBsAg clearance rate, the acute HBV DNA clearance rate, the HBsAg and HBV DNA rebound rates at 24 h post-treatment. The safety endpoints included an incidence of adverse events/serious adverse events or fluctuations in the key blood components (TP and ALB) pre- vs. post-treatment.

### 2.15. Statistical Analysis

GraphPad Prism 8.0 software was used for statistical analyses, as described in the figure legends. For a comparison of the parametric data between two groups, a two-tailed Student’s *t*-test was applied. For comparisons of the parametric data among three or more groups, one-way ANOVA was used with Tukey’s post hoc test. All biochemical and cellular assays were performed in at least three independent replicates, and representative results are shown. Statistical significance was defined as follows: *** *p* ≤ 0.001 indicates extremely significant, ** *p* ≤ 0.01 indicates highly significant, * *p* ≤ 0.05 indicates statistically significant, and *p* > 0.05 implies no statistically significant difference.

## 3. Results

### 3.1. Preparation and Sequence Characterization of Murine Anti-HBsAg mAb

**Figure 1:**
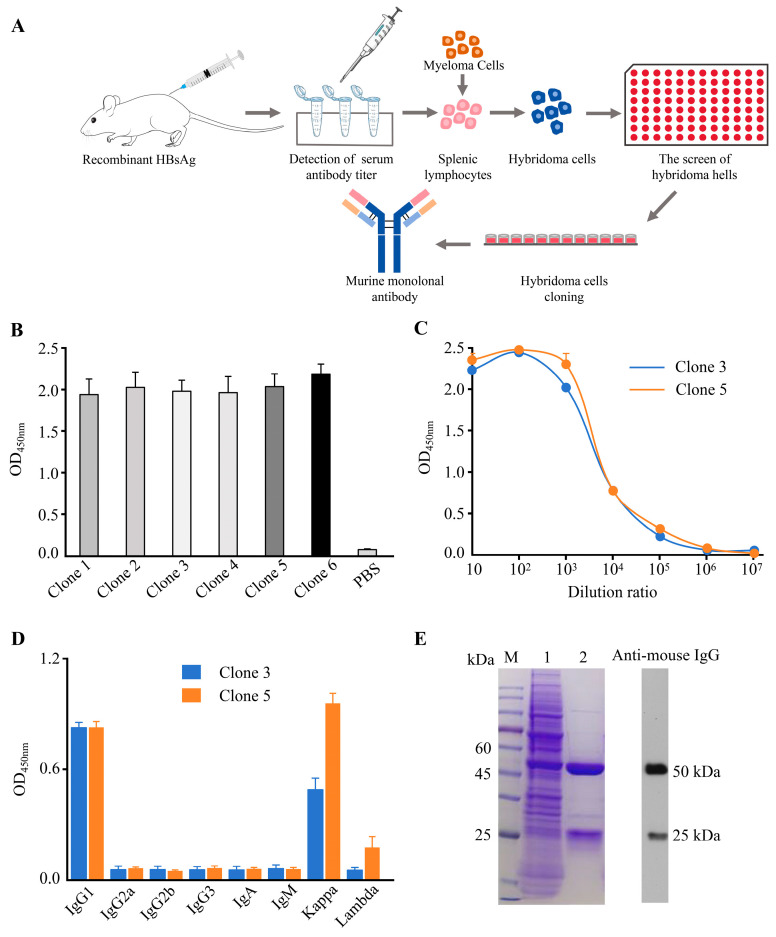
The preparation and characterization of murine anti-HBsAg monoclonal antibodies (mAbs). (**A**) A schematic flowchart of murine anti-HBsAg mAb preparation. BALB/c mice were immunized with recombinant hepatitis B virus surface antigen (HBsAg). Specific antibodies were subsequently screened using hybridoma technology. (**B**) Screening of hybridoma subclones. Antibodies in the supernatant of hybridoma cell lines were detected by an indirect enzyme-linked immunosorbent assay (iELISA). Six antibody-positive subclones (Clone 1–Clone 6) exhibiting stronger binding affinity to HBsAg were identified based on OD_450nm_ measurements. Data shown are mean ± SD from three replicates. (**C**) Titer analysis of antibodies from Clone 3 and Clone 5 hybridoma cell culture supernatants. Supernatant antibodies were subjected to 10-fold serial dilutions (1:10 to 1:10^7^), and antibody titers were quantified by measuring OD_450nm_ values. Data shown are mean ± SD from three replicates. (**D**) Isotype identification of antibodies from Clone 3 and Clone 5 supernatants. The antibody isotypes present in the hybridoma culture supernatants were determined using a commercially available antibody isotyping kit. Data shown are mean ± SD from three replicates. (**E**) SDS-PAGE (left) and Western blotting analysis (right) of Clone 5 antibody purified from mouse ascites. Lane M: Prestained protein molecular weight ladder; Lane 1: Proteins after caprylic acid-ammonium sulfate precipitation; Lane 2: Antibody purified by Protein A affinity chromatography. The secondary antibody used for WB was HRP-conjugated goat anti-mouse IgG.

Murine mAbs against HBsAg were generated by immunizing BALB/c mice with recombinant HBsAg, followed by hybridoma technology, as outlined in Figure 1A. Screening of the hybridoma culture supernatants via iELISA identified six positive subclones (Clone 1–Clone 6) based on OD_450nm_ readings (Figure 1B). However, only Clones 3 and 5 exhibited stable growth upon scale-up in 6-well plates. An antibody titer analysis revealed that supernatants from Clones 3 and 5 retained effective binding to recombinant HBsAg antigens even at a dilution of 1:100,000 (1:10^5^) (Figure 1C), indicative of high affinity. Isotyping confirmed that mAbs from both clones belonged to the IgG1 heavy-chain subclass with kappa light chains (Figure 1D). Ascitic fluid generated via the intraperitoneal injection of Clone 5 hybridoma cells into mice was subjected to initial antibody enrichment via caprylic acid-ammonium sulfate precipitation. Reducing the SDS-PAGE analysis of the precipitate showed multiple diffuse bands, reflecting the complexity of proteins in murine ascites (Figure 1E, Lane 1). Following resolubilization of the precipitate, purification via Protein A affinity chromatography yielded distinct bands corresponding to the heavy chain (approximately 50 kDa) and light chain (approximately 25 kDa) under reducing SDS-PAGE conditions (Figure 1E, Lane 2), thus clearly demonstrating structural integrity of the antibody. To validate the antibody specificity, a Western blotting analysis was performed using an HRP-conjugated goat anti-mouse IgG antibody. Specific signals were detected at the expected molecular weights (50 kDa and 25 kDa), confirming the immunoreactivity of both the heavy and light chains of the murine antibody.

The sequencing of the Clone 3 and 5 hybridoma cell lines revealed identical VH and VL chain sequences for the murine anti-HBsAg mAb. The VH coding sequence was 348 bp, encoding 116 amino acids, and the VL coding sequence was 321 bp, encoding 107 amino acids (the sequences are provided in Supplementary Table S1). The framework regions (FRs) exhibited high conservation, while the CDRs displayed high variability, responsible for antigen binding. According to the IMGT numbering scheme, the VH amino acid sequence comprised FR1 (1-25)-CDR1 (26-33)-FR2 (34-50)-CDR2 (51-58)-FR3 (59-96)-CDR3 (97-105)-FR4 (106-116). The VL amino acid sequence comprised FR1 (1-26)-CDR1 (27-32)-FR2 (33-49)-CDR2 (50-52)-FR3 (53-88)-CDR3 (89-97)-FR4 (98-107).

### 3.2. Humanization of Murine Anti-HBsAg mAb

**Figure 2:**
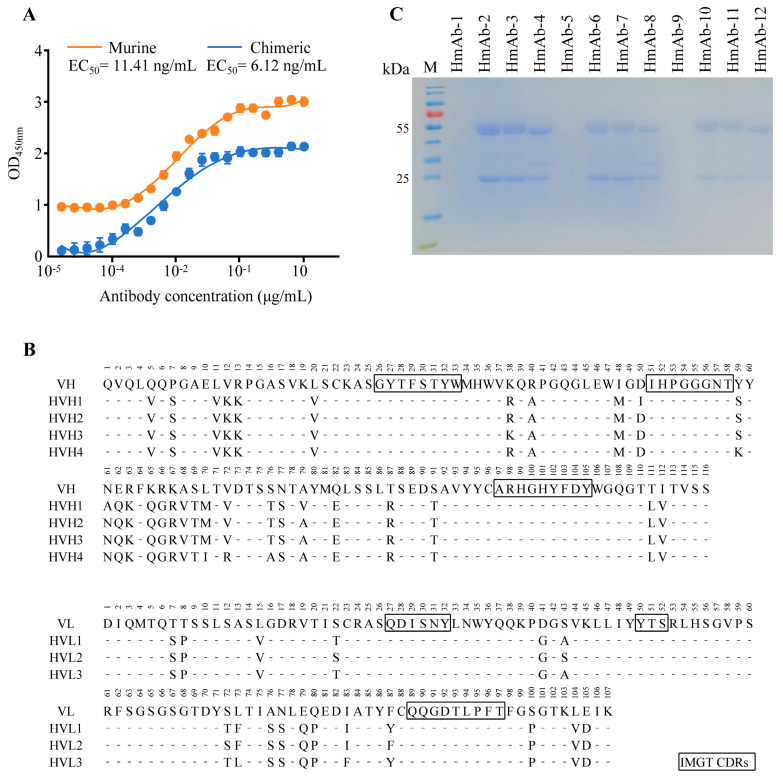
The humanization of murine anti-HBsAg mAbs. (**A**) The determination of half-maximal effective concentration (EC_50_) for murine and chimeric antibodies. EC_50_ values were calculated using curve fitting analysis in GraphPad Prism^®^ software. Data represent mean ± SD (n = 3). (**B**) Sequence alignment analysis of candidate humanized variants. Four candidate heavy chain variable region sequences (HVH1-HVH4) and three candidate light chain variable region sequences (HVL1-HVL3) were designed using the Molecular Operating Environment (MOE 2019.0102) software platform. (**C**) SDS-PAGE analysis of candidate humanized antibodies. Twelve humanized monoclonal antibodies (HmAb-1 to HmAb-12) were generated by combinatorial pairing of the designed variable regions. Purified antibodies from supernatants of transiently transfected HEK293E cells were resolved under reducing conditions. Lane M: Prestained protein molecular weight marker; Lanes 1–12: HmAb-1 to HmAb-12 samples.

To verify the HBsAg-binding activity of the murine VH/VL sequences, the VH and VL genes were cloned into the eukaryotic expression vector pTT5 containing the constant domains of human immunoglobulin, constructing human–mouse chimeric antibody expression plasmids pTT5-VH and pTT5-VL, respectively (the sequences are provided in Supplementary Table S2). The transient transfection of HEK293E cells yielded the chimeric antibody. The purified chimeric antibody exhibited an EC_50_ value of 6.12 ng/mL when binding to recombinant HBsAg (Figure 2A), comparable with the EC_50_ of the parental murine antibody (11.41 ng/mL), confirming the retention of a favorable antigen-binding capacity. Consequently, this VH/VL sequence served as the template for humanization.

Four humanized VH sequences (HVH1-HVH4) and three humanized VL sequences (HVL1-HVL3) were obtained using the MOE platform (Figure 2B). Twelve humanized antibodies (HmAb-1 to HmAb-12) were generated via combinatorial pairing of these chains, with the pairing details provided in Supplementary Table S3. The corresponding gene fragments were cloned into eukaryotic expression vectors and transiently transfected into HEK293E cells using PEI, and the antibodies were purified from culture supernatants. A reducing SDS-PAGE analysis revealed the absence of intact IgG bands for HmAb-1, HmAb-5, and HmAb-9 (Figure 2C), while the other nine antibodies (HmAb-2/3/4/6/7/8/10/11/12) were successfully expressed. An analysis of the binding activity in the cell supernatants revealed EC_50_ values of 622.20 ng/mL, 404.95 ng/mL, and 596.99 ng/mL for HmAb-1, HmAb-5, and HmAb-9, respectively, indicating severely compromised antigen-binding capacity. In contrast, the other nine antibodies maintained high affinity, with EC_50_ values ranging from 0.41 ng/mL to 13.89 ng/mL (Supplementary Figure S1). These results were fully consistent with the SDS-PAGE data.

The affinity of purified antibodies was assessed using SPR and iELISA (Table 1). All nine successfully expressed humanized antibodies exhibited *K*_D_ in the range of 10^−9^ to 10^−10^ M, meeting the criteria for high affinity. Notably, HmAb-4, HmAb-8, and HmAb-12 demonstrated *K*_D_ values of 10^−10^ M, approximately one order of magnitude lower than those of the other antibodies. Despite its high affinity (*K*_D_ = 0.35 nM), HmAb-4 was excluded from further development due to its substantially higher EC_50_ (821.65 ng/mL), indicating inferior functional potency. Notably, all three high-affinity antibodies contained HVH4, whereas the non-expressing HmAb-1/5/9 all featured HVH1. A sequence alignment (Figure 2B) suggested that the D50I and N61A mutations within the heavy-chain CDR2 region likely caused expression or folding defects in HmAb-1/5/9. The iELISA analysis revealed superior binding potency for HmAb-8 (EC_50_ = 35.53 ng/mL) and HmAb-12 (EC_50_ = 79.39 ng/mL). Transient expression quantification demonstrated significantly higher antibody production in HmAb-12 (80.03 ± 10.72 mg/L) compared with HmAb-8 (33.35 ± 7.23 mg/L) (Supplementary Figure S2). Based on these findings, HmAb-12 was selected as the lead candidate for stable transfection.

### 3.3. Establishment and Functional Characterization of Stable Cell Lines

The HVL3 and HVH4 genes encoding HmAb-12 were cloned into mammalian expression vectors containing a glutamine synthetase (GS) selection marker and designated pGS-CMV-HVL3 and pGS-CMV-HVH4, respectively (vector schematics in Supplementary Figure S3). Following transfection into CHO-K1 cells via electroporation, stable pools were selected under 25 μM MSX pressure. Single-cell printing facilitated the isolation of 2880 clones, from which the top five high-expressing monoclonal lines (ssc-A to ssc-E) were identified.

**Figure 3:**
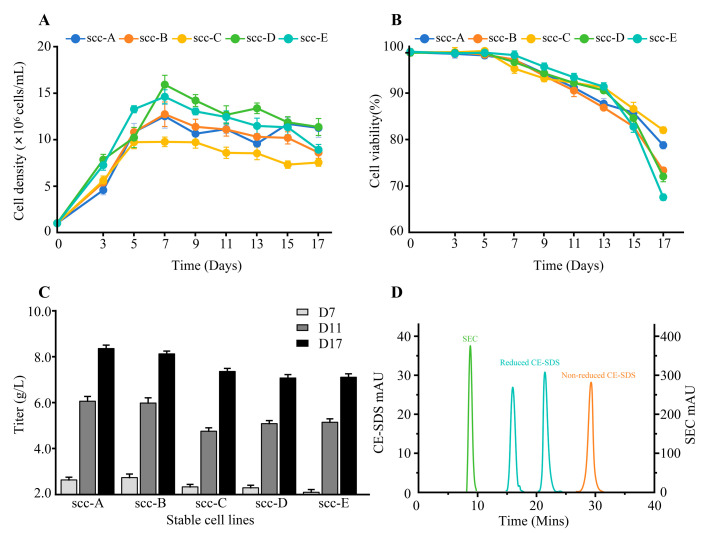
Analysis of cell growth, antibody production, and product quality in 17-day fed-batch culture. (**A**,**B**) Cell density and viability monitoring during fed-batch cultures of CHO-K1 cells. Cell lines scc-A to scc-E represent five distinct monoclonal stable clones. (**C**) Secreted HmAb-12 antibody titers measured on days 7, 11, and 17. (**D**) Purity analysis of HmAb-12 antibody harvested from scc-A fed-batch cultures on day 17. Size exclusion chromatography (SEC) and capillary electrophoresis-sodium dodecyl sulfate (CE-SDS) were employed for antibody purity assessment. SEC-HPLC (x-axis): Retention time (Minutes); CE-SDS (x-axis): Migration time (Minutes). Data represent mean ± SD (n = 3).

Fed-batch cultures of the ssc-A to ssc-E clones demonstrated progressive increases in antibody expression over 17 days (Figure 3C), with concurrent monitoring of viable cell density and viability (Figure 3A,B). Notably, ssc-A and ssc-C maintained superior viability (78.70% and 81.93%, respectively) and high viable cell densities at the endpoint. The harvest titers reached 8.31 g/L (ssc-A), 8.08 g/L (ssc-B), 7.32 g/L (ssc-C), 7.04 g/L (ssc-D), and 7.07 g/L (ssc-E).

The purification of fed-batch supernatants via Protein A affinity chromatography revealed exceptional product quality across all top clones (Table 2). The SEC-HPLC analysis confirmed a monomeric purity of >97% for all lines, with ssc-A achieving 99.24% (Figure 3D). CE-SDS under reducing conditions showed purity between 96.74% and 98.70% (ssc-A highest at 98.70%), while non-reduced CE-SDS showed purity ranging from 96.75% to 97.78%. An integrated assessment identified ssc-A as the optimal producer line based on its superior titer (8.31 g/L), monomer content (99.24%), and CE-SDS purity. This cell line was subsequently deposited at the China Center for Type Culture Collection (accession: CCTCC NO: C202492).

### 3.4. Preparation of an HmAb-12-Based Immunoadsorbent

**Figure 4:**
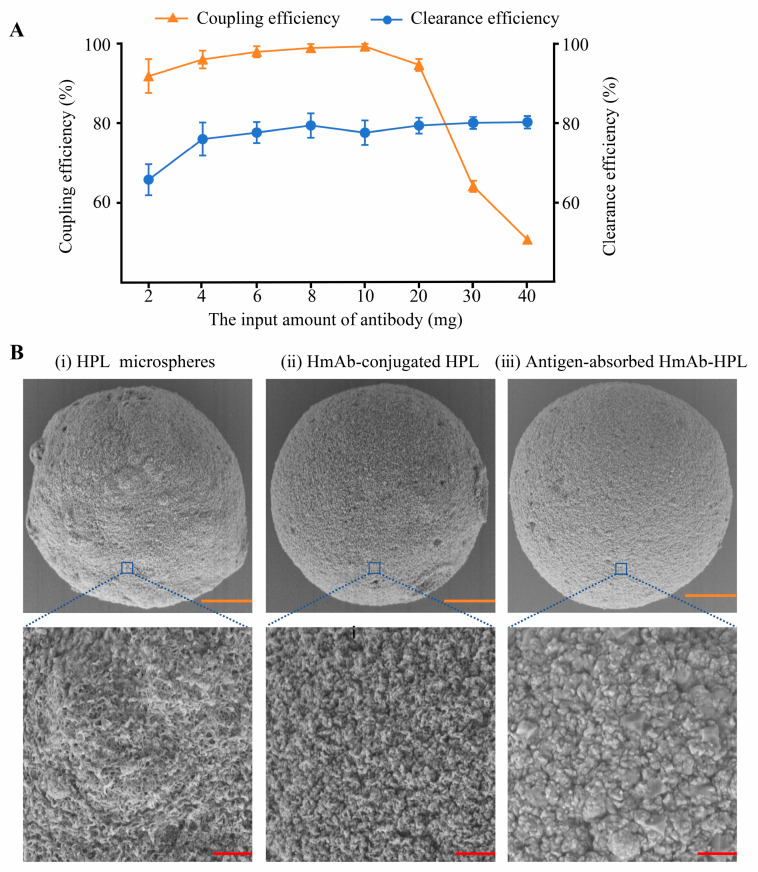
The preparation of HBsAg-specific immunoadsorbents. (**A**) The coupling efficiency of HmAb-12 (left Y-axis) and HBsAg clearance efficiency of corresponding immunoadsorbents (right Y-axis) versus immobilized HmAb-12 dosage. Data represent mean ± SD (n = 3). (**B**) Scanning electron microscopy (SEM) analysis of microspheres. Representative SEM images show: (**i**) unmodified HPL microspheres, (**ii**) antibody-conjugated microspheres, and (**iii**) antigen–antibody complex-bound microspheres. Yellow scale bars = 10 μm; red scale bars = 1 μm. Images are representative of three independent replicates.

The humanized mAb HmAb-12 was covalently conjugated to Focurose HPL agarose microspheres via sodium periodate oxidation. Figure 4A illustrates the relationship between antibody loading density (2–40 mg/g), coupling efficiency, and HBsAg clearance. The results demonstrate that within the 4–40 mg/g loading range, the adsorbent achieved consistent recombinant HBsAg clearance (75–80%). Based on a cost–benefit analysis, 4 mg/g was selected as the optimal loading density. SEM characterization (Figure 4B) revealed the following: (i) Sodium periodate-activated HPL beads exhibited characteristic three-dimensional porous networks, providing optimal surface area for antibody immobilization. (ii) Surface morphology became markedly denser with continuous antibody coverage through multipoint anchoring, confirming effective covalent fixation. (iii) Immune complexes completely occupied the residual pores, forming continuous biointerfaces with reduced surface roughness and partial smoothening, demonstrating specific antigen–antibody binding-induced topological alterations.

**Figure 5:**
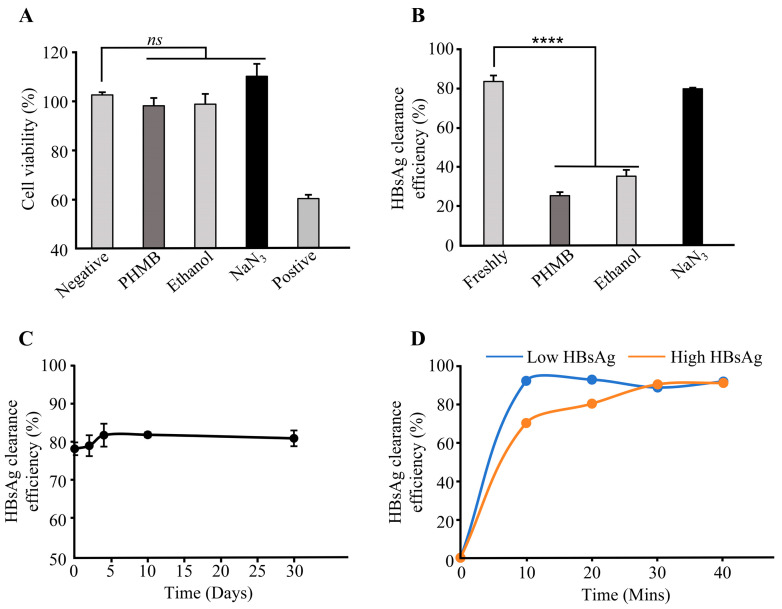
The performance evaluation of HBsAg-specific immunoadsorbents. (**A**) Cytotoxicity assessment of preservative-treated immunoadsorbents by MTT assay. Negative control: Polyethylene extract; Positive control: 5% DMSO; Test groups: 0.4 g/L PHMB, 20% (*v*/*v*) ethanol, 1 g/L NaN_3_. Data were analyzed using a two-tailed independent Student’s *t*-test. A *p*-value > 0.05 was considered not statistically significant (ns). (**B**) Effect of storage solutions on HBsAg clearance efficiency at 4 °C. Immunoadsorbents were equilibrated at 4 °C in: freshly prepared solution (no preservative), 0.4 g/L PHMB, 20% (*v*/*v*) ethanol, or 1 g/L NaN_3_. Two-tailed unpaired Student’s *t*-test was performed (**** *p* < 0.0001). (**C**) Accelerated stability testing. Clearance efficiency for recombinant HBsAg was measured after storage at 37 °C for 0, 2, 4, 10, and 30 days. (**D**) Dynamic adsorption efficiency in vitro. Immunoadsorbents packed in 5 mL columns underwent recirculating adsorption with bovine serum containing varying HBsAg concentrations. Residual HBsAg levels were quantified at 10, 20, 30, and 40 min to calculate clearance efficiency. Data represent mean ± SD (n = 3).

To evaluate the preservative effects on adsorbent performance, three storage solutions were compared: polyhexamethylene biguanide hydrochloride (PHMB), ethanol, and sodium azide (NaN_3_). MTT assays were used to demonstrate that >98% of L929 cells remained viable across all groups (Figure 5A), confirming negligible cytotoxicity. After 30-day storage at 4 °C, the NaN_3_-preserved adsorbent maintained 79.65% antigen clearance, comparable to the rate for the freshly prepared adsorbent (83.36%) and significantly superior to those for the PHMB (25.24%) and ethanol (35.15%) groups (Figure 5B). The performance degradation induced by PHMB likely stems from the synergistic effects of its cationic properties: (1) Guanidinium oligomers competitively occupy antibody paratopes. (2) Zeta potential shifts disrupt antigen–antibody electrostatic interactions, whereas ethanol, as a polar organic solvent, may denature HBV antibody conformation, thereby impairing binding capacity and reducing adsorbent performance. Consequently, NaN_3_ was selected for further evaluation. Accelerated stability testing (37 °C) showed 80.99% clearance after 30 days (Figure 5C), with no statistically significant difference (*p* > 0.05) from the thermal equilibrium baseline (78.28%), validating robust storage stability under NaN_3_.

The dynamic in vitro circulation adsorption results are presented in Figure 5D. Under low-antigen-load conditions, the adsorbent achieved 92.10% HBsAg clearance after just a single processing cycle, confirming its rapid binding kinetics for HBsAg. Under high-antigen-load conditions, cumulative clearance progressively increased from 70.14% (Cycle 1) to 80.23% (Cycle 2), reaching 90.28% after three cycles, confirming its superior dynamic adsorption capacity. Notably, the marginal increase to 91.02% in Cycle 4 suggests near-saturation adsorption under high viral loads.

### 3.5. Preclinical Feasibility Trial

**Figure 6:**
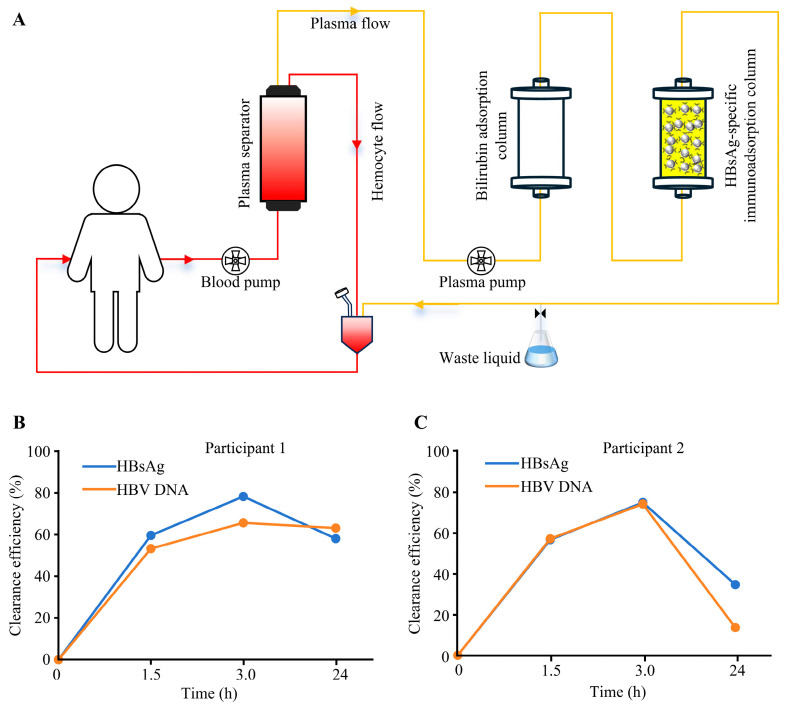
The clinical therapeutic efficacy of the HBsAg-specific immunoadsorption column. (**A**) A schematic of the extracorporeal treatment system. (**B**) Viral marker kinetics in a high-antigen-load participant (baseline HBsAg: 80,000 IU/mL). (**C**) Viral marker kinetics in an intermediate-antigen-load participant (baseline HBsAg: 3000 IU/mL). Data points represent single measurements.

The HBsAg-specific immunoadsorption column (HBV-250) was evaluated for safety and efficacy in two CHB participants. Participant 1 presented a high antigen load (baseline HBsAg: 80,000 IU/mL), while participant 2 exhibited a moderate antigen load (baseline HBsAg: 3000 IU/mL). A tandem extracorporeal circulation system (Figure 6A) was used to perform dual-column plasma adsorption with processed volumes of 1.8–2.5 L.

Viral clearance kinetics (Figure 6B,C) demonstrated that after the first cycle, HBsAg clearance reached 59.64% in participant 1 and 56.20% in participant 2, with parallel reductions in HBV DNA of 53.27% and 56.69%, respectively. Following the second cycle, clearance efficiency significantly increased: HBsAg removal rose to 78.30% (participant 1) and 74.31% (participant 2), while HBV DNA clearance reached 65.66% and 73.55%, respectively. The highly concordant kinetics of both viral markers indicate the effective capture of subviral particles and intact virions. At 24 h post-treatment, the high-antigen-load participant (participant 1) exhibited rebound rates of 20.20% for HBsAg and 2.52% for HBV DNA. In contrast, the moderate-antigen-load participant (participant 2) demonstrated rebound rates of 39.94% (HBsAg) and 60.09% (HBV DNA). These results confirm viral rebound for both markers in all participants, with significantly lower rebound rates in the high-antigen-load participant versus the moderate-load Participant.

Monitoring of key blood components revealed less than 15% variation in total protein and albumin levels post-treatment, with fluctuations remaining under 5% at 24 h and values returning to physiological ranges (Table 3). No serious adverse events were recorded.

## 4. Discussion

The development of novel therapeutic strategies to achieve sustained HBsAg clearance remains a pivotal challenge in managing HBV-associated liver disease. This study successfully developed a high-affinity humanized anti-HBsAg mAb (HmAb-12; *K*_D_ = 0.36 nM) and engineered it into a targeted immunoadsorption system. In a preliminary evaluation involving two participants, this system was associated with a reduction in HBsAg of approximately 75% and in HBV DNA of more than 65%.

In this study, we engineered a humanized antibody through the integration of homology-based modeling prediction, CDR grafting, and framework residue point-mutation optimization. This strategy successfully preserved high antigen affinity while reducing immunogenicity, achieving results on par with established antibody humanization approaches [32,33]. The CHO-K1 stable cell line developed through high-throughput screening achieved an antibody titer of 8.31 g/L under fed-batch culture conditions, significantly exceeding conventional CHO production systems. This enhanced productivity is attributed to synergistic codon optimization and the GS/MSX selection system [34], effectively addressing scalability limitations in therapeutic antibody manufacturing.

The therapeutic value of hemoadsorption technology in viral infectious diseases continues to gain prominence. Lectin-functionalized adsorption columns selectively clear free virions from patient plasma by targeting mannose residues on Ebola virus particles, achieving viral load reductions below detection limits within 6 days post-treatment and ultimately facilitating patient recovery [35]. In chronic hepatitis C therapy, the double-filtration plasmapheresis regimen combined with PEG-IFNα and ribavirin physically removes hepatitis C virus particles and core protein complexes from plasma through small-pore filters, enhancing antiviral efficacy and restoring immune cell function [36]. The affinity hemodialysis system incorporating HIV-specific anti-gp120 antibodies achieves ≈90% viral clearance in vitro using both cell culture supernatants and whole blood, providing an alternative therapeutic approach for patients with drug resistance or immune reconstitution failure [37]. Cellulose acetate/metal–organic framework composite microbeads achieve 99.93% HIV capture efficiency during extracorporeal hemoperfusion through synergistic porous architecture and hydrogen bonding [38]. Heparin-coated polyethylene beads effectively clear pathogens from the bloodstream by mimicking the heparan sulfate-binding mechanism on endothelial cell surfaces, targeting acute liver failure induced by HSV-2 and EBV infections [39]. Based on the specific binding mechanism between the SARS-CoV-2 spike protein and human angiotensin-converting enzyme 2 (hACE2), the SARS-catch column with immobilized hACE2 peptides can specifically capture the virus, shortening the duration of ventilator weaning in critically ill patients [40]. The above-mentioned studies have confirmed the significant clinical value of blood adsorption technology in the treatment of viral infections. Our data show that this immunoadsorption system can effectively remove HBsAg within a short time frame (3 h, Figure 6B,C), supporting its further investigation as a potential complementary approach for managing conditions related to acute HBV infection.

Regarding the treatment of CHB induced by HBV, previous studies have demonstrated that patients with low-level HBsAg (<100 IU/mL) exhibit sustained HBV-specific immune responses and HBsAg seroconversion following the discontinuation of NAs [41,42]. This phenomenon indicates that reduced circulating HBsAg levels facilitate a clinical cure for CHB, thereby underscoring the pivotal role of HBsAg clearance [43]. In the present study, a single plasmapheresis adsorption cycle achieved >50% HBsAg clearance, with efficacy further increased to >75% following a second cycle. These preliminary clinical findings demonstrate the effective clearance of HBsAg by the specific adsorption column, suggesting its potential relevance for the clinical management of CHB. The current pharmacologic management of CHB confronts major clinical challenges, including suboptimal HBsAg seroclearance rates and virologic rebound following treatment cessation [44,45]. Although HBsAg seroclearance (functional cure, FC) signifies clinical control of HBV, persistent cccDNA often remains in hepatocyte nuclei as a stable transcriptional reservoir resistant to current antivirals like NAs [46]. Nevertheless, achieving FC significantly reduces intrahepatic cccDNA levels and HBV integration in CHB patients [47,48]. Specifically, studies demonstrate that 27% of functionally cured patients (13/48) achieve complete cccDNA clearance, while the majority (73%) retain trace amounts at levels 20-fold lower than baseline [48]. This reduction reflects combined effects of immune-mediated silencing (e.g., histone deacetylation) and antiviral-driven degradation (e.g., natural degradation after replication inhibition), highlighting that functional cure does not universally eradicate cccDNA but minimizes its transcriptional activity. In this small-scale study (n = 2), a rebound in HBsAg and HBV DNA levels was noted within 24 h post-treatment. Although no firm conclusions can be drawn due to the limited sample, the observed disparity in rebound kinetics-where the participant with a moderate viral load exhibited higher rebound rates than the one with a high viral load-warrants further investigation in larger studies. This differential response likely originates from divergent viral replication kinetics: under high-viral-load conditions, the expanded intrahepatic cccDNA reservoir and prolonged antigen synthesis cycle collectively result in slower viral replenishment. Conversely, moderate-viral-load participant exhibit increased cccDNA transcriptional activity, accelerating viral particle re-synthesis and thereby inducing significant rebound. To mitigate post-treatment viral rebound, we propose combinatorial strategies leveraging synergies between immunoadsorption and established antivirals. The adsorption column achieves rapid HBsAg reduction (>70% clearance per cycle), creating an antigen-low milieu that may potentiate NAs efficacy through sustained viral polymerase inhibition, thereby blocking de novo virion synthesis. Complementarily, the antigen nadir post-adsorption provides an optimal window for interferon immunomodulation, wherein PEG-IFNα administration may reverse T-cell exhaustion to enhance sustained immune control in low-antigen environments, as evidenced by reduced rebound in participants achieving HBsAg < 100 IU/mL [41,42].

In the management of HBV-related acute-on-chronic liver failure (HBV-ACLF), non-bioartificial liver support systems (NBALSSs) constitute essential extracorporeal organ replacement interventions. By deploying modalities including plasma exchange (PE), plasma diafiltration, and the double-plasma molecular adsorption system (DPMAS), NBALSSs effectively eliminate toxins, thereby establishing a critical therapeutic window for hepatic regeneration or bridging to transplantation [49,50]. Clinical evidence demonstrates that combining PE with the DPMAS significantly enhances the 12-week survival rates in HBV-ACLF patients, with improvements reaching up to 28% [51]. A separate investigation revealed that the DPMAS with integrated PE (DPMAS + PE) combined with antiviral agents achieved substantial HBV-DNA reduction by day 7 of therapy. This early virologic response may stem from synergistic interactions between antiviral suppression and PE-mediated viral load dilution. Notably, the DPMAS + PE regimen demonstrated superior sustained virologic suppression at day 90 compared to PE monotherapy [52]. Nevertheless, current NBALSSs suffer from an inherent deficiency in pathogen-directed specificity during toxin clearance, thereby constraining their therapeutic efficacy. In contrast to the non-selective toxin adsorption of conventional NBALSSs, the HBsAg-specific immunoadsorption column engineered in this study enables high-affinity capture and elimination of key viral components, specifically HBsAg and HBV DNA. This study successfully constructed a efficient adsorption column that integrates high-affinity antibody engineering with a blood purification platform. The stable and high-yield antibody expression may lead to reduced production costs. Given its demonstrated compatibility with existing NBALSSs, this technology represents a candidate worthy of further investigation for combinatorial therapeutic regimens in advanced liver failure management.

Nevertheless, this study has several limitations: (1) Although humanized antibody engineering mitigates immunogenicity risks, the long-term safety implications of trace-level antibody leakage require further assessment through primate models. (2) Given the small sample size (n = 2) and short follow-up (24 h), these results should be interpreted as proof-of-concept. Long-term multi-cycle trials are needed to assess sustained viral suppression and clinical outcomes. The limited cohort size and single-intervention evaluation may constrain statistical robustness. (3) A systematic analysis of the kinetic impact of blood HBsAg clearance on intrahepatic cccDNA dynamics and HBsAg reservoir depletion remains to be addressed.

## 5. Conclusions

Integrating antibody engineering with immunoadsorption technology, this study establishes a novel precision strategy for targeted HBV elimination. Preliminary results from a limited cohort (n = 2) indicate its potential for efficient viral antigen clearance with a favorable initial safety profile, showing approximately 75% acute HBsAg reduction. The establishment of a high-yield (8.31 g/L) production process supports its potential for further development. However, the preliminary nature of these findings necessitates further validation. Future work will focus on evaluating long-term safety in primate models, assessing multi-cycle efficacy in combination with nucleos(t)ide analogs (NAs) and pegylated interferon alpha (PEG-IFNα), and exploring its role in personalized therapeutic protocols for chronic hepatitis B.

## Figures and Tables

**Table 1:** The affinity measurement of the candidate humanized anti-HBsAg monoclonal antibody.

	Candidate Humanized mAb									Murine mAb
	HmAb-2	HmAb-3	HmAb-4	HmAb-6	HmAb-7	HmAb-8	HmAb-10	HmAb-11	HmAb-12	
*K*_D_ (nM)	1.95	4.52	0.35	2.01	3.64	0.30	3.04	7.07	0.36	1.87
EC_50_ (ng/mL)	281.52	367.81	821.65	188.99	1182.11	35.53	255.25	976.94	79.39	1.68

**Table 2:** Product quality attributes of top-performing humanized anti-HBsAg mAbs in fed-batch culture.

	SEC Data	CE-SDS Data	
	Main Peak (%)	Reduced Purity (%)	Non-Reduced Purity (%)
scc-A	99.24	98.70	97.09
scc-B	98.97	96.74	97.61
scc-C	99.09	96.97	96.75
scc-D	99.21	97.12	97.24
scc-E	97.28	97.28	97.78

**Table 3:** Changes in serum total protein and albumin levels in participants before and after treatment.

Participant	Parameter (g/L)	Pre-Treatment	Post-Treatment (0 h)	Change Rate (%) *	Post-Treatment (24 h)	Change Rate (%) *
Participant 1	TP	65.60	57.70	−12.04	67.20	2.44
	ALB	43.20	37.90	−12.27	42.80	−0.93
Participant 2	TP	71.80	61.80	−13.93	68.50	−4.60
	ALB	41.90	36.40	−13.13	41.00	−2.15

TP: Total protein (reference range: 65–85 g/L). ALB: Albumin (reference range: 40–55 g/L). * Change rate = [(Post-value − Pre-value)/Pre-value] × 100%.

## Data Availability

Data are contained within this article.

## Supplementary Materials

The following supporting information can be downloaded at https://www.mdpi.com/article/10.3390/biomedicines13092175/s1, Table S1: Amino acid sequences of the heavy- and light-chain variable regions of the murine anti-HBsAg mAb; Table S2: Expressed amino acid sequence of chimeric antibody; Table S3: Pairing combinations of candidate humanized VH/VL; Figure S1: Diagram of the stable expression vector containing the target gene. Figure S2: Transient expression quantification of HmAb-8 and HmAb-12. Figure S3: Diagram of the stable expression vector containing the target gene.
