# Supplementary material for: Performance Evaluation of a HBsAg-Specific Immunoadsorbent Based on a Humanized Anti-HBsAg Monoclonal Antibody
Source: Biomedicines. 2025 Sep 5;13(9):2175. doi: 10.3390/biomedicines13092175 (PMC12467161; doi:10.3390/biomedicines13092175)

Supplementary Table S1: Amino acid sequences of the heavy- and light-chain variable regions of the murine anti-HBsAg mAb.

		IMGT
Variable region of heavy chain	QVQLQQPGAELVRPGASVKLSCKASGYTFS TYWMHWVKQRPGQGLEWIGDIHPGGGNT YYNERFKRKASLTVDTSSNTAYMQLSSLTSE DSAVYYCARHGHYFDYWGQGTTITVSS	CDR-H1: GYTFSTYW CDR-H2: IHPGGGNT CDR-H3:ARHGHYF DY
Variable region of light chain	DIQMTQTTSSLSASLGDRVTISCRASQDISNY LNWYQQKPDGSVKLLIYYTSRLHSGVPSRF SGSGSGTDYSLTIANLEQEDIATYFCQQGDT LPFTFGSGTKLEIK	CDR-L1: QDISNY CDR-L2: YTS CDR-L3:QQGDTLP FT

Supplementary Table S2: Expressed amino acid sequence of chimeric antibody.

pTT5-VH	MKHLWFFLLLVAAPRWVLSQVQLQQPGAELVRPGASVKLSCKASGYTFSTY WMHWVKQRPGQGLEWIGDIHPGGGNTYYNERFKRKASLTVDTSSNTAYMQ LSSLTSEDSAVYYCARHGHYFDYWGQGTTITVSSASTKGPSVFPLAPSSKSTS GGTAALGCLVKDYFPEPVTVSWNSGALTSGVHTFPAVLQSSGLYSLSSVVTV PSSSLGTQTYICNVNHKPSNTKVDKKVEPKSCDKTHTCPPCPAPELLGGPSVF LFPPKPKDTLMISRTPEVTCVVVDVSHEDPEVKFNWYVDGVEVHNAKTKPR EEQYNSTYRVVSVLTVLHQDWLNGKEYKCKVSNKALPAPIEKTISKAKGQP REPQVYTLPPSREEMTKNQVSLTCLVKGFYPSDIAVEWESNGQPENNYKTTP PVLDSDGSFFLYSKLTVDKSRWQQGNVFSCSVMHEALHNHYTQKSLSLSPG K*
pTT5-VL	MVLQTQVFISLLLWISGAYGDIQMTQTTSSLSASLGDRVTISCRASQDISNYL NWYQQKPDGSVKLLIYYTSRLHSGVPSRFSGSGSGTDYSLTIANLEQEDIATY FCQQGDTLPFTFGSGTKLEIKRTVAAPSVFIFPPSDEQLKSGTASVVCLLNNFY PREAKVQWKVDNALQSGNSQESVTEQDSKDSTYSLSSTLTLSKADYEKHKV YACEVTHQGLSSPVTKSFNRGEC*

Supplementary Table S3: Pairing combinations of candidate humanized VH/VL.

	HVH1	HVH2	HVH3	HVH4
HVL1	HmAb-1	HmAb-2	HmAb-3	HmAb-4
HVL2	HmAb-5	HmAb-6	HmAb-7	HmAb-8
HVL3	HmAb-9	HmAb-10	HmAb-11	HmAb-12

Supplementary Figure S1: Evaluation of antigen-binding activity: EC50 determination of antibodies purified from culture supernatants of transiently transfected HEK293E Cells

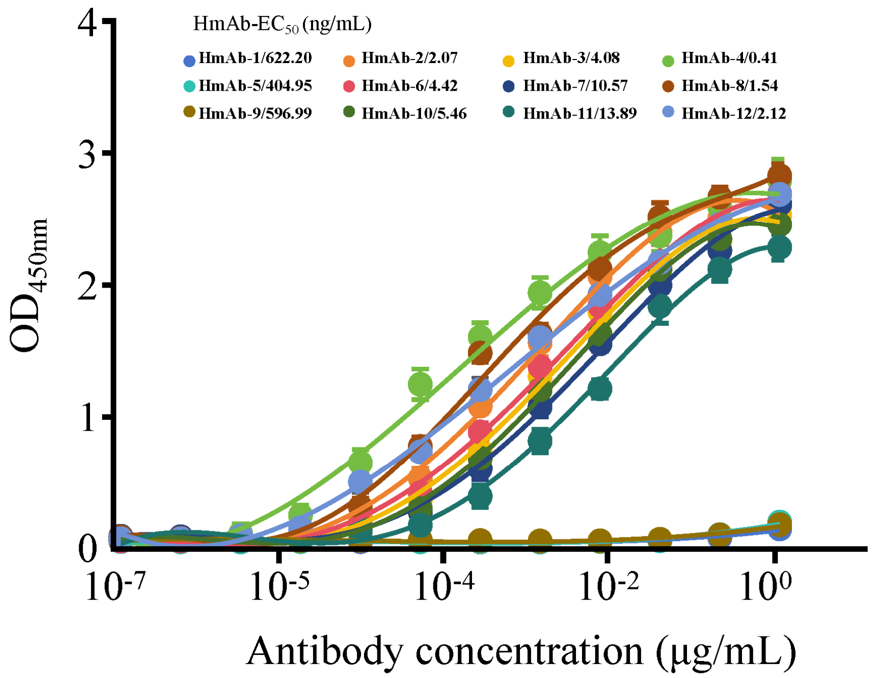

The 12 antibody samples (HmAb-1 - HmAb-12) were purified from culture supernatants using affinity chromatography. The antigen-binding activity of the purified antibodies was then assessed using an indirect ELISA (iELISA). The absorbance at 450 nm (OD_{450}) was measured for each dilution using a microplate reader. EC_{50} values were calculated using curve fitting analysis in GraphPad Prism software. HmAb-1/622.20 indicates that the EC_{50} value of HmAb-1 antibody is 622.20 ng/mL. Data represent mean ± SD (n = 3).

Supplementary Figure S2: Transient expression quantification of HmAb-8 and HmAb-12.

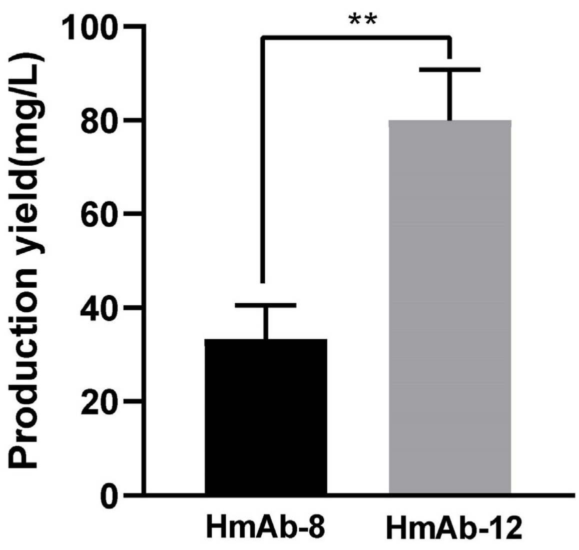

293E cells were transiently transfected with the corresponding plasmids, and the antibody expression level in the supernatant was measured. Experiments were repeated three times. Data were analyzed by two-tailed unpaired Student's *t*-test (**p < 0.01).

Supplementary Figure S3: Diagram of the stable expression vector containing the target gene.

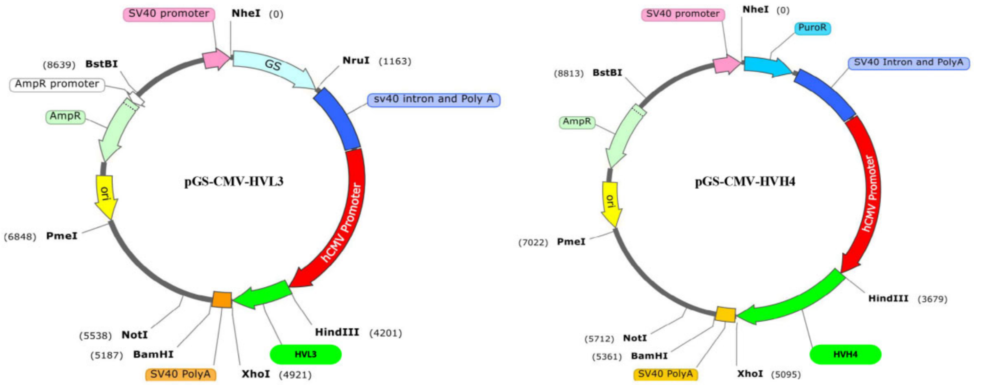

Supplement: Supplementary file 1 [file biomedicines-13-02175-s001.zip › biomedicines-3816290-supplementary.pdf]
